# The Actin Binding Protein Adseverin Regulates Osteoclastogenesis

**DOI:** 10.1371/journal.pone.0109078

**Published:** 2014-10-02

**Authors:** Siavash Hassanpour, Hongwei Jiang, Yongqiang Wang, Johannes W. P. Kuiper, Michael Glogauer

**Affiliations:** 1 Matrix Dynamics Group, Faculty of Dentistry, University of Toronto, Toronto, Ontario, Canada; 2 Department of Operative Dentistry and Endodontics, Guanghua School of Stomatology, Sun Yat-sen University, Guangdong Provincial Key Laboratory of Stomatology, Guangzhou, P. R. China; University of Oulu, Finland

## Abstract

Adseverin (Ads), a member of the Gelsolin superfamily of actin binding proteins, regulates the actin cytoskeleton architecture by severing and capping existing filamentous actin (F-actin) strands and nucleating the assembly of new F-actin filaments. Ads has been implicated in cellular secretion, exocytosis and has also been shown to regulate chondrogenesis and megakaryoblastic leukemia cell differentiation. Here we report for the first time that Ads is involved in regulating osteoclastogenesis (OCG). Ads is induced during OCG downstream of RANK-ligand (RANKL) stimulation and is highly expressed in mature osteoclasts. The D5 isoform of Ads is not involved in regulating OCG, as its expression is not induced in response to RANKL. Three clonal Ads knockdown RAW264.7 (RAW) macrophage cell lines with varying degrees of Ads expression and OCG deficiency were generated. The most drastic OCG defect was noted in the clonal cell line with the greatest degree of Ads knockdown as indicated by a lack of TRAcP staining and multinucleation. RNAi mediated knockdown of Ads in osteoclast precursors resulted in distinct morphological changes characterized by altered F-actin distribution and increased filopodia formation. Ads knockdown precursor cells experienced enhanced migration while fusion of knockdown precursors cells was limited. Transient reintroduction of de novo Ads back into the knockdown system was capable of rescuing TRAcP expression but not osteoclast multinucleation most likely due to the transient nature of Ads expression. This preliminary study allows us to conclude that Ads is a RANKL induced early regulator of OCG with a potential role in pre-osteoclast differentiation and fusion.

## Introduction

The bone extracellular matrix (ECM) has historically been described as a static and protective scaffold [1]. Yet in reality, bone ECM is subjected to periodical remodeling to maintain its strength and integrity [2], [3]. The task of skeletal remodeling falls in the domain of osteoclasts, which degrade the inorganic and organic phases of bone [1] and osteoblasts, which produce and secrete new matrix and regulate matrix mineralization [4]. Under normal circumstances bone destruction and formation are in steady state equilibrium. However, imbalances in bone remodeling result in perturbations of skeletal structure, integrity and function leading to diseases such as osteoporosis [5], osteopetrosis [6], inflammatory osteolysis such as rheumatic arthritis, periodontal disease [3], [7] and Paget's bone disease [3]. Even though bone remodeling requires the collaborative action of osteoblasts and osteoclasts, the common thread to all the aforementioned disorders is abnormal bone resorption. Therefore, a thorough understanding of osteoclast formation or osteoclastogenesis (OCG) is crucial for development of novel drugs for treating bone-related diseases.

Osteoclasts are tissue specific multinuclear cells derived from hematopoietic stems cells [8] of the macrophage/monocyte lineage [9]. The intricate process of OCG, which involves coordinated cellular migration [10], [11], adhesion and membrane fusion [12]– is regulated by the critical hematopoietic cytokines, Macrophage Colony Stimulating Factor (M-CSF) and Receptor Activator of NF-κB Ligand (RANKL) [15]. OCG also requires dynamic regulation of cellular actin cytoskeleton. Cells organize their actin cytoskeleton through interactions with actin binding proteins [16]–[18] that control the length, flexibility and the viscosity of the actin network leading to changes in cell morphology and function. One such group of actin binding proteins is the Gelsolin superfamily, consisting of seven highly conserved members [17]. Adseverin (Ads), also known as Scinderin, is the closest homologue to the founding member of the Gelsolin superfamily. Like Gelsolin, Ads can promote F-actin depolymerization and nucleation depending on the intracellular conditions [19]. However, unlike Gelsolin, Ads's F-actin severing activity is inhibited by a wider range of membrane lipids [20]–[22], and its activation requires lower intracellular calcium concentrations [23]. Ads has been heavily implicated in regulating exocytosis through a rapid depolymerization of cortical actin in a number of biological systems, including chromaffin cells [24], airway goblet cells [25], murine pancreatic β-cell [26] and platelets [27]. In addition, Ads has been identified as a member of a multi-protein complex required for the trafficking of the water channel aquaporin-2 in rat renal collecting ducts [28] and has been shown to play a role in the differentiation of platelets [29] and chondrocytes [30]. A report by Robbens et al. [31] noted that Interleukin-9 stimulated T-helper lymphocytes express a splice variant of Ads, Ads D5, that is missing most of the fifth and a portion of the sixth Gelsolin like domains. Ads D5 has most of the typical characteristic common to all members of the Gelsolin family of actin binding proteins, with the exception of nucleation of filament assembly *in vitro*.

Surprisingly very little is known about the role of Ads during OCG. Unlike Gelsolin, which regulates osteoclast function and motility [32]–[34], a microarray analysis published by Yang et al. [35] is the only existing publication identifying Ads as a gene of interest in osteoclasts. A similar microarray performed in our lab also identified Ads as a gene of interest, which led us to test the hypothesis that Ads is a RANKL induced regulator of OCG with a potential functional role during osteoclast formation and function. The present study focused on investigating the role of Ads in OCG using two established *in vitro* model systems. It was shown for the first time that Ads is expressed during OCG in response to soluble RANKL (sRANKL) at both transcript and protein levels. Several Ads knockdown (KD) clonal cell lines with varying degrees of Ads expression reduction were generated. The clonal KD cell line with the greatest reduction in Ads expression failed to undergo OCG upon treatment with sRANKL, a phenotype most likely caused by a defect in osteoclast precursor fusion. The attenuation of Ads led to distinct morphological changes characterized by altered F-actin remodeling. The reintroduction of Ads was capable of rescuing the pre-osteoclast differentiation in the KD cells, in the form of TRAcP expression. Therefore, we concluded that Ads is a novel RANKL induced regulator of OCG with a functional role during the early stages of OCG.

## Materials and Methods

### 2.1 Cell cultures

All procedures described were performed in accordance with the Guide for the Humane Use and Care of Laboratory Animals and were approved by the University of Toronto Animal Care Committee. Bone marrow monocytes (BMMs) were isolated from 6–12-week-old wild-type (WT) mice (SV129/BL6) as previously described [36]. OCG was induced by seeding 3×10^6^ cells onto 60-mm^2^ culture dishes in the Minimum Essential Medium – Alpha (α-MEM) (Gibco Life Technologies, Cat. No. 11095) containing 10% Fetal Bovine Serum (FBS) and 164 IU/mL of penicillin G, 50 µg/mL of gentamicin, and 0.25 µg/mL of fungizone (complete media), supplemented with 20 ng/ml M-CSF (Sigma, Cat No. M9170) for two days (unstimulated cells) or with 20 ng/ml M-CSF plus 30 ng/ml of sRANKL (Peprotech, Cat. No. 315-11) for up to 6 days. Fresh culture media supplemented with cytokines was added to the cells every two days. Cells were cultured at 37°C (5% CO_2_). Murine RAW264.7 (RAW) macrophages (ATCC, provided by Keyin Li and Morris F Manolson at the University of Toronto) were cultured in the complete Dulbecco's Modified Eagle's Medium (DMEM) (Gibco Life Technologies, Cat. No. 11995). For osteoclast differentiation, 2.5×10^5^ cells were seeded in 6-well culture dishes and cultured with 30 ng/ml sRANKL for up to 4 days. Unstimulated RAW cells were used as controls. Cells were cultured at 37°C (constant high humidity and 5% CO_2_). All RAW cells used were between passage 5 and 12 to minimize the effects of passage on cell differentiation.

### 2.2 Microarray analysis

Total bone marrow cells from three mice were harvested, mixed and stimulated for 48 hours in complete α-MEM supplemented with 10 ng/ml M-CSF. Non-adherent cells were collected and centrifuged at 350×g for 30 minutes at 22°C over Ficoll-Paque PLUS (GE Healthcare, Cat No. 17-1440-02). A layer of mononuclear osteoclast precursors was obtained and split into two dishes, each containing 8×10^5^ cells/cm^2^. One dish was stimulated with 20 ng/ml M-CSF plus 200 ng/ml of purified sRANKL (as previously described [36]), and the other with 20 ng/ml M-CSF. After two days, the cells were washed with α-MEM and the total RNA was extracted (RNeasy Mini Kit, Qiagen, Cat. No. 74104). The quality of total RNA was determined using Agilent Technologies BioAnalyser. Microarray was performed using Affymetrix GeneChip Mouse Gene 1.0 ST array. Three independent experiments were performed making cells from three mice a single biological repeat. Gene expression profiles of cytoskeleton-associated genes were filtered. Gene normalization was performed prior to subsequent clustering computation using MultiExperiment Viewer 4.9.0 (MeV -TM4) [37]. MeV-HCL was run by defining Pearson Correlation metrics and an Average Linkage clustering method. Only genes with the statistically significant changes in expression of at least two-fold were reported. The data discussed in this publication have been deposited in NCBI's Gene Expression Omnibus and are accessible through GEO Series accession number GSE54779 (http://www.ncbi.nlm.nih.gov/geo/query/acc.cgi?acc=GSE54779).

### 2.3 Quantitative real-time PCR

Total RNA was extracted and residual genomic DNA was eliminated (RNase-Free DNase Kit, Qiagen, Cat. No. 79254). RNA concentration was determined using the Thermo Scientific Nanodrop 1000 Spectrophotometer. Ten random samples were analyzed for RNA quality using the Agilent Technologies Bioanalyzer. One µg of RAW and 120 ng BMM total RNA was reverse transcribed into cDNA using 200 units of Superscript II reverse transcriptase (Invitrogen, Cat. No. 18064-022) and 1 µM Oligo-dT_18_ VN primers (ACGT Corp). A 1:32 cDNA dilution was used for all primer pairs to yield optimal PCR efficiency. Quantitative real-time PCR was performed in triplicate using the BioRad CFX96 real-time system. Each 20 µL reactions contained: 5 uL of 1:10 diluted cDNA, 500 nM of forward and reverse primers and 10 µL of SsoFast EvaGreen Supermix (Bio-Rad, Cat No. 172-5200). The PCR conditions were as follows:

95°C 2 min followed by 40 cycles of 95°C for 5 sec and 60°C/65°C for 5 sec. Melt curve analysis (95°C for 5 sec, 65°C for 5 sec, and 65° to 95°C with 0.5°C increase every 5 sec) of the amplified product was used to determine the specificity of the PCR reaction. The CFX Manger Software (Version 1.0) was used to analyze the PCR results. mRNA expression was normalized to the control, internal housekeeping gene GAPDH [38]. The following primers were used: *Ads* F: 5′-CCTATGGTGACTTTTACGTCGG-3′, R: 5′-CTCATCCTGGGAACACTCCTT-3′; *GAPDH* F: 5′-CCTTCCGTGTTCCTACCCC-3′, R: 5′-GCCCAAGATGCCCTTCAGT-3′; *Gelsolin* F: 5′-CCTACCGCACATCCCCCAG-3′, R: 5′-GTGATGGGGGTCCGCCTG-3′, *RANK* F: 5′-CTAATCCAGCAGGGAAGCAAAT-3′, R: 5′-GACACGGGCATAGAGTCAGTTC-3′; *SIRPα* F: 5′-TCGAGTGATCAAGGGAGCAT-3′, R: 5′-CCTGGACACTAGCATACTCTGAG-3′ and *CD44* F: 5′-TAGGAGAAGGTGTGGGCAG-3′, R: 5′-AGGCACTACACCCCAATC-3′.

### 2.4 Western blot analysis

Immunoblotting was carried as previously described [36]. Equal amounts of total cell lysates (TCL) were resolved via SDS-PAGE on 8–12% polyacrylamide gels and transferred onto a nitrocellulose membrane (GE Healthcare, Cat. No. 8549062). Non-specific sites were blocked with Tris-buffered saline plus 0.05% v/v Tween 20 (TBS-T) with 5% nonfat milk powder for 1 hour. Incubations with primary antibodies were carried out overnight (O/N) at 4°C and secondary antibody for 1 hour at room temperature (RT). The following antibodies were used: Rabbit polyclonal anti-murine-Ads antibody (gift from Dr. C. Svensson [39], 1:2,000); Rabbit polyclonal anti-murine-Gelsolin antibody (gift from Dr. C. McCulloch, 1:2,000); Mouse monoclonal anti-murine ß-actin (Sigma, Cat No. A5316, 1:8,000); Rabbit polyclonal anti-murine Cathepsin K antibody (Abcam Inc, Cat No. ab19207, 1:1000); -conjugated donkey anti-rabbit IgG (GE Healthcare, Cat. No. NA934V); Sheep anti-mouse IgG-HRP (Amersham Pharmacia Biotech; Cat. No. NA931V). Immuno-reactive proteins were detected using chemiluminescence with Amersham ECL Plus Western Blotting Detection System (GE Healthcare, Cat. No. RPN2232), upon exposure to Bioflex MSI film (Clonex Corporation, Cat. No. CLMS810). Films were developed using the Kodak M35A X-OMAT Processor, scanned digitally using the Epson Perfection 1250 scanner and band intensities were quantified by densitometry using NIH ImageJ 1.41 software. Results are expressed as relative expression versus internal loading control, ß-actin.

### 2.5 Detection of Adseverin isoforms

Primers were designed to flank the fifth domain (D5) of Ads (esD5-F: 5′-GTGCAGGTCCGTGTCTCTC-3′, esD5-R: 5′-GTGCAGGTCCGTGTCTCTC-3′). The design of the primers allowed for the specific detection of both Ads isoforms. Specifically, if the full-length Ads isoform was expressed, a 650 bp product was expected. However, if the smaller, Ads D5 isoform was expressed, a 350 bp product was expected (Fig. S1). Taq DNA polymerase (Qiagen, Cat. No. 201203) was used to non-quantifiably amplify the targeted sequences using cDNA from RAW macrophage and BMM-derived osteoclasts. PCR using 10 pg of pSE380-Ads and pSE380-Ads D5 (kind gift from Dr. J. Robbens [31]), and esD5 primers yielded 650 bp and 350 bp products respectively, thus allowing for the differentiation of two isoforms. To check for protein expression of Ads isoforms, 20 µg of TCL from unstimulated RAW and BMMs as well as TCL from Day 6 BMM cultures and Day 4 RAW cultures were resolved on an SDS-PAGE gel and immunoblotted against Ads. Lysates from BL21 E. coli (Agilent Technologies, Cat. No. 200132) transformed with pSE380-Ads or pSE380-Ads D5 and induced with 1 mM IPTG were used as positive controls. Two distinct bands at 85 kDa and 80 kDa are readily identifiable in lysates of BL21 *E. coli* induced to express exogenous Ads or Ads D5, respectively.

### 2.6 Retroviaral shRNA knockdown construct preparation

Ads specific (underlined) single stranded oligonucleotides were designed in accordance to the guidelines described by the Clontech RNAi systems (top strand: 5′-GATCCAACAAATATGAGCGTCTGATTCAAGAGATCAGACGCTCATATTTGTTTTTTTTACGCGTG-3′, bottom strand: 5′-AATTCACGCGTAAAAAAAACAAATATGAGCGTCTGATCTCTTGAATCAGACGCTCATATTTGTTG-3′). The complementary oligonucleotides were annealed and ligated into the linearized RNAi-Ready-pSIREN-RetroQ-DsRed-Express vector (Clontech, Cat No. 632487) using T4 DNA ligase (New England Biolabs, Cat. No. M0202L) for 1 hour at RT. In addition, a separate ligation was carried out using the manufacturer provided Luciferase (Luc, underlined) control oligonucleotides (top strand 5′-GATCCGTGCGTTGCTAGTACCAACTTCAAGAGATTTTTTACGCGTG-3′, bottom strand 5′-AATTCACGCGTAAAAAATCTCTTGAAGTTGGTACTAGCAACGCACG-3′). The ligated construct was used to transform competent DH5 *E. Coli* (Invitrogen, Cat. No. 44-0098). The transformed *E. Coli* were allowed to recover for 1 hours at 37°C while shaking at 250 rpm. The transformed cultures were plated onto Ampicillin (100 µg/ml) selective media and incubated O/N at 37°C. A bacteria colony was selected and used to inoculate 2 mL of LB/Amp culture media, and incubated at 37°C for 8 hours while shaking at 250 rpm. The shRNA construct was extracted (Qiagen, QIAprep spin miniprep kit, Cat. No. 27108) and the DNA concentration was determined using the Nanodrop spectrophotometer. A fraction (1 µg) of the construct was digested with 20 U of MluI (New England Biolabs, Cat. No. R0198L) to confirm the presence of the insert. The sequence of the insert was confirmed by sequencing (ACGT Corp, Toronto, ON).

### 2.7 shRNA delivery and characterization of knockdown cell lines

GP-293 pantrophic packaging cells (gift from Drs. Helen Sarantis and Scott D. Gray-Owen, Department of Molecular and Medical Genetics, University of Toronto) were co-transfected (FuGENE HD, Roche, Indianapolis, Cat. No. 04709691001) with 2 µg of shRNA retroviral constructs (Ads-shRNA, Luc-shRNA) as well as 2 µg of the pVSV-G envelope protein-packaging vector (gift from Drs. Helen Sarantis and Scott D. Gray-Owen). Transfected GP-293 cells were incubated at 37°C for three days at which point viral containing supernatant was harvested and spun free of cells. The viral supernatant was filtered (Millex-HA 0.45 µm, Millipore, Cat. No. SLHA033SS), and residual cellular DNA degraded (Benzonase, Sigma, Cat. No. E1014), prior to RAW cell infections. Infected RAW cells were incubated for three days at 37°C and sorted based on the DsRed signal by fluorescence-activated cell sorting, using a Beckman-Coulter flow cytometer. 8.5×10^5^ and 1.0×10^6^ of Luc KD and Ads KD macrophages were sorted. DsRed positive cells were subjected to a standard limiting dilution. Briefly, 130 cells were resuspended in 6.5 mL cell culture media to get 2 cells/100 µL. One hundred µL cell suspension was added into each well of row A, B and C of a 96-well flat-bottomed plate. The remained cells were further diluted with 2.9 mL cell culture media to give a concentration of 1 cell/100 µL. One hundred µL cell suspension was added into each well of row D, E and F. Finally, the remained cells were further diluted with 2.2 mL cell culture media to reach 0.5 cell/100 µL, theoretically. One hundred µL of the cell suspension was seeded into each well of row G and H of the above plate. After one week incubation, colonies were examined under an inverted fluorescent microscope at 20× objective magnification and DsRed positive colonies were identified and expanded. Three Ads KD clonal cell lines (Ads KD Clone B, Clone C, Clone F), one Luc KD clonal cell line, as well as, non-infected WT RAW macrophages were expanded. A schematic outlining the generation of the various cell lines can be found in Fig. S2.

### 2.8 TRAcP staining and osteoclastogenesis index

BMMs and RAW macrophages were seeded in 6-well tissue culture plates at 2.5×10^5^ cells/well and OCG was induced via 6-day and 4-day stimulations with sRANKL, respectively. Cells were washed twice with Phosphate Buffer Saline (PBS), fixed with 4% paraformaldehyde (PFA) and stained for tartrate-resistant acid phosphatase (TRAcP) as previously described [40]. Briefly, fixed cells were incubated in a solution of naphthol AS-BI phosphate and fast red TR salt in 0.2 M acetate buffer (pH 5.2) containing 100 mM sodium tartrate (Sigma, Cat No. S-4797) for 30 min at 37°C and washed with PBS. Cells were viewed with a Leitz Wetzlar microscope and images taken with a PixeLinK camera. The OCG index was determined by quantifying the number and size of TRAcP-positive osteoclasts in 10 random fields of view (FOVs) at 20× objective magnification. TRAcP positive osteoclasts with 3–10 nuclei were considered to be small osteoclasts, whereas TRAcP positive osteoclasts with 10–30, and 30+ nuclei were considered to be medium and large osteoclasts, respectively. The fusion index was quantified in a similar manner.

### 2.9 Differential seeding density assay

To evaluate the effect of initial cell seeding density on OCG, WT and KD cell lines were seeded onto 8-well slide chambers at three initial seeding densities: 1×10^4^ cells/well (low density), 5×10^4^ cells/well (normal density) and 1.0×10^5^ cells/well (high density). Day 4 cultures were fixed and TRAcP stained as described above. The degree of osteoclast formation was determined by viewing strained cells at 20× objective magnification and representative images were taken with a PixeLink camera.

### 2.10 Transwell migration Assay

Two million cells were stimulated with sRANKL (60 ng/mL) in 10 mL of the complete DMEM for 30 hours in the 10-cm^2^ Petri dishes. The cells were sRANKL starved O/N. The cells were scrapped and counted using a hemocytometer and resuspended in the reduced DMEM (0.5% FBS and antibiotics) to a final concentration was 1×10^6^ cells/mL. Migration assays were carried out as previously described [41]. The transwells (8 µm) were pre-equilibrated and placed in the wells of a 24-well plate containing 600 µL reduced DMEM plus 60 ng/mL sRANKL. Two hundred microliters of cell suspension (containing 2×10^5^ cells) were seeded into each well. The cells were incubated for 20 hours to allow for migration. After migration, non-migratory cells on the top of the membrane were removed with cotton swabs. The cells on the bottom of the membrane were fixed with 4% PFA and labeled with 0.165 µM 4′,6′-diamidino-2-phenylindole hydrochloride (DAPI) in PBS/0.1% Triton-100. The pictures were taken by using a Nikon Eclipse E1000 microscope. The experiment was repeated three times.

### 2.11 Functional resorption assay

WT, Luc KD and Ads KD cells were cultured for up to 6 days (+60 ng/mL sRANKL) in 96-well culture plates or 12-well cultures plates containing 18-CIR coverglass (Fisherbrand) or dentin slices prepared from narwhal tusks [42]. Cells cultured on the glass were fixed (4% PFA) and TRAcP stained. Pictures were taken by using a Nikon Eclipse E1000 microscope. To view resorption pits, cells were removed from the dentine slices by a 5 minute treatment in 6% NaOCl. The slices were painted with 1% toluidine blue (wt/vol) and 1% sodium borate (wt/vol) for 30 sec. Discs were viewed and representative images taken using a Nikon Elipse E400 microscope.

### 2.12 F-actin localization using confocal microscopy

Cells were cultured with sRANKL for two days. Cell culture medium was aspirated, cells were washed twice with pre-warmed PBS and permeabilized with 0.1% Triton-100 in PBS. Non-specific binding was blocked for 30 min at RT with 1% BSA/0.1% Triton-100/PBS. The cells were incubated in 100 mM glycine solution for 10 min and incubated in dark for 20 min in Alexa Fluor 488 phalloidin (0.15 µM in 1% BSA/0.1% Triton-100/PBS). A z-stack of 70 sections confocal images was taken (Leica, DMIRE2). A section at the glass was shown for each cell and the 3D reconstruction was construction using the maximizing projection method. Fluorescent intensity of two representative cells was measured by ImageJ. The number of membrane protrusions was enumerated in 30 representative cells, for each cell type respectively, and results are expressed as mean (+/- SD).

### 2.13 Adseverin rescue using site directed mutagenesis

As previously mentioned in the methods section 2.6, a 19 bp region of the Ads open reading frame (5′- ^nt. 547^ AACAAATATGAGCGTCTGA ^nt. 565^-3′) was targeted by the Ads specific shRNA. Using site-directed mutagenesis a new, mutated, Ads open reading frame was constructed where the same 19 bp region reads as follows: 5′- ^nt. 547^ AACAA**G**TA**C**GA**A**CG**G**CT**C**A ^nt. 565^-3′. These nucleotide substitutions (A^552^-G, T^555^-C, G^558^-A, T^561^-G, G^564^-C) lead to five silent mutations such that the final amino acid sequence of both the original and mutated sequences was the same (N-K-Y-E-R-L). The newly constructed mut-Ads was cloned into the BamHI and BstBI restrictions site of pIRESpuro3 vector (Clontech, Mountain View, CA). Two million Ads KD Clone F cells were transiently transfected with 2 µg of pIRESpuro3-mut-Ads using Amaxa nucleatransfector and solution V (Program D-032). One fraction was stimulated for 4 days with sRANKL (60 ng/ml) and the cells were harvested for Western blot analysis. The other fraction was stimulated for 5 days with sRANKL (60 ng/ml) and the cells were processed for TRAcP staining.

### 2.14 Statistical analysis

For experiments where multiple observations were made per sample, numerical results were expressed as mean ± SEM or mean ± SD, as indicated. Each experiment had a sample size of n≥3, unless otherwise stated. Statistical analysis was performed using Student *t*-test and a *p* value of less than 0.05 was considered statistically significant.

## Results

### 3.1 Adseverin expression is up-regulated during RANKL-induced OCG

**Figure 1:**
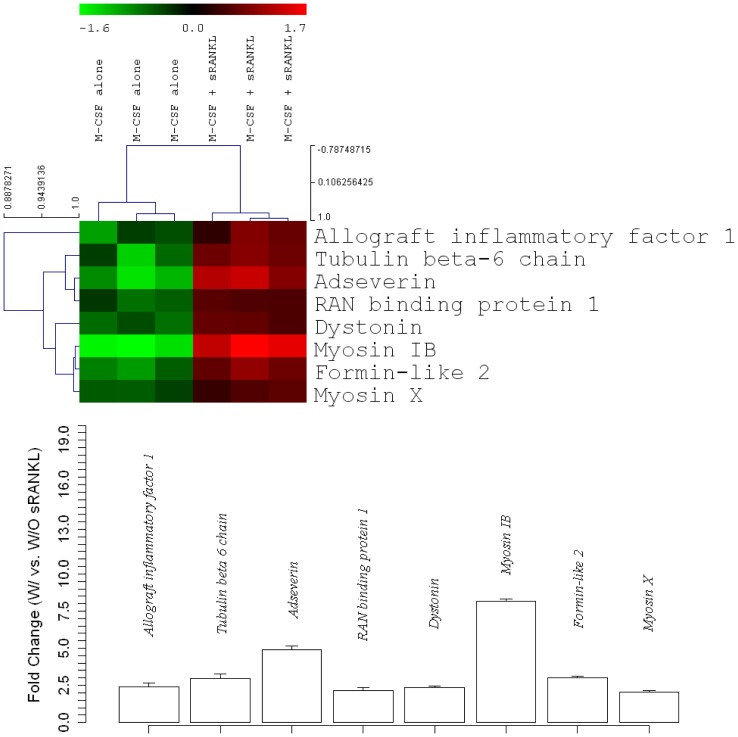
Microarray analysis identified Adseverin as a gene of interest during the early phases of OCG. Several genes were differentially expressed in osteoclast precursors stimulated for two days with sRANKL & M-CSF versus M-CSF alone. Eight cytoskeleton-associated genes with the greatest fold-change in expression (p<0.05) were reported. Specifically, Ads was up-regulated 4.9-fold in osteoclast precursors stimulated with sRANKL for two days. The microarray data has been deposited in NCBI's Gene Expression Omnibus and are accessible through GEO Series accession number GSE54779.

An exploratory microarray was carried out to identify RANKL regulated genes during OCG. This microarray analysis (unpublished data) identified eight cytoskeleton associated genes of interest with statistically significant changes in gene expression of least 2-fold in BMMs stimulated for two days with M-CSF and sRANKL versus M-CSF alone (Fig. 1). Of these genes, Ads showed the second greatest change in expression with a 4.9-fold increase in expression in response to sRANKL. This finding prompted us to closely examine the temporal expression pattern of Ads during OCG.

**Figure 2:**
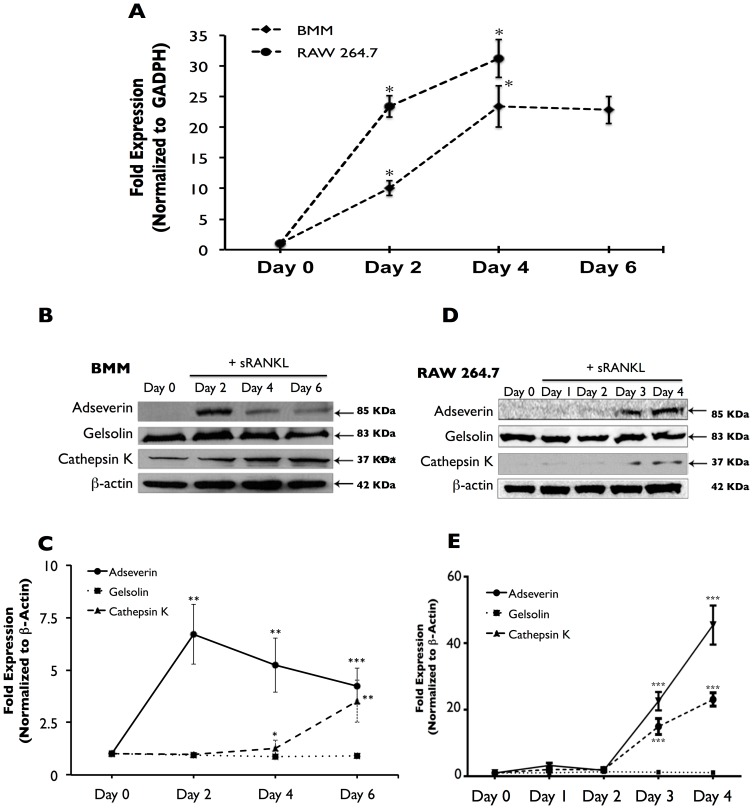
Adseverin expression is up-regulated during OCG in response to sRANKL. **A**) Quantitative real-time PCR analysis was used to quantify Ads gene expression in osteoclast cultures derived from BMMs (black diamonds) and RAW macrophages (black circles). Results are expressed as fold expression versus Day 0 and are normalized against GADPH ± SEM. In BMM-derived osteoclasts, Ads expression was significantly up-regulated by 10-fold after 2 days and 23-fold after 4 days. No difference was noted between Day 4 and Day 6 cultures. In RAW-derived osteoclasts, Ads gene expression was significantly up-regulated by 24-fold after 2 days and 32-fold after 4 days. **B**) Immunoblot analysis was used to quantify protein expression in BMMs and RAW macrophage-derived osteoclast cultures. Results are expressed as fold expression versus Day 0 and normalized against ß-actin ± SD. In BMM-derived osteoclasts, Ads protein expression was significantly up-regulated by 6-fold in Day 2 cultures. 5-fold and 4-fold increases in expression were noted in Day 4 and 6 cultures, respectively. The decrease in expression in days 4 and Day 6 was not found to be statistically significant. Gelsolin expression was not altered during OCG in response to sRANKL. Cathepsin K expression was significantly increased in response to sRANKL after a 4 day stimulation with sRANKL. **D**) Similarly in RAW macrophage-derived osteoclast cultures, Ads was up-regulated 17-fold and 23-fold in Day 3 and Day 4 cultures respectively. Gelsolin expression was not altered during OCG, and Cathepsin K expression was significantly increased in Day 3 and 4 cultures. **C & E**) Quantification of immunoblots (* p<0.05, ** p<0.01, *** p<0.001, n = 3).

In BMM-derived osteoclasts, Ads transcript was significantly up-regulated by 10-fold in Day 2 osteoclasts compared to basal Ads transcript expression in Day 0 BMMs. Day 4 cultures showed a significant 13-fold increase in expression when compared to Day 2 cultures. No statistically significant difference was noted between transcript levels of Day 4 and Day 6 cultures, both of which had 23-fold higher expression compared to Day 0 BMMs (Fig. 2A, black diamonds). RAW macrophage-derived osteoclasts also experienced significant 23 and 31-fold up regulation in Ads transcript in Day 2 and Day 4 cultures compared to Day 0 macrophages (Fig. 2A, black circles). The transcript levels were significantly higher in Day 4 cultures than in Day 2 cultures.

In BMM-derived osteoclasts, Ads protein expression was significantly up-regulated by 6-fold in Day 2 cultures versus Day 0 cultures. Ads protein expression remained elevated during osteoclast maturation. Ads protein expression in Day 4 and 6 cultures did not statistically differ from Day 2 (Fig. 2B & 2C). In RAW-derived osteoclast cultures (Fig. 2D & 2E), Ads protein was not detected in Day 1 or Day 2 cultures, while statistically significant 17 and 23-fold up regulation were noted in Day 3 and Day 4 cultures. Ads protein levels were significantly greater in Day 4 cultures compared to Day 3 cultures. In both model systems, Gelsolin protein levels did not change in response to sRANKL, while the expression osteoclast marker gene Cathepsin K was significantly increased by Day 4 in BMM cultures (Fig. 2B & 2C) and Day 3 in RAW macrophage cultures (Fig. 2D & 2E).

### 3.2 Adseverin D5 is not expressed during OCG

**Figure 3:**
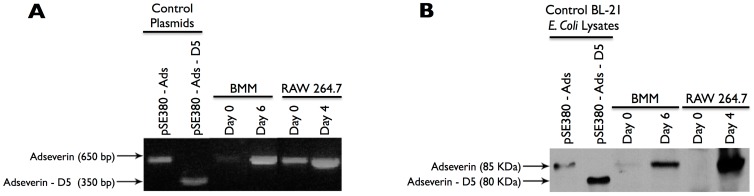
Adseverin D5 is not is expressed during *in vitro* OCG. **A**) PCR detected only the full-length Ads isoform in Day 0 and Day 6 BMMs and Day 0 and Day 4 RAW macrophage cultures. pSE380-Ads (full-length Ads) and pSE380-Ads-D5 (Ads D5 isoform) plasmids were used as positive controls. Equal volumes of cDNA from each representative samples were used as templates. The intensity of the signal is not a quantitative measure of Ads gene expression. **B**) Immunoblot analysis also failed to detect the Ads D5 in BMM and RAW macrophage-derived osteoclasts. Lysates from the transformed BL21 *E. coli* induced to express exogenous protein were used as positive controls. Only the 85 kDa full-length isoform was detected in 20 µg of TCL from Day 6 BMMs and Day 4 RAW macrophages cultures. Ads was not detected in 20 µg of TCL from unstimulated samples.

PCR analysis only detected the 650 bp product, representing full-length Ads, in Day 0 RAW macrophages and BMM as well as Day 4 RAW and Day 6 BMM-derived osteoclasts (Fig. 3A). This observation was validated at the protein level where only the 85 KDa full-length Ads was detected in lysates of mature osteoclasts derived from RAW macrophages and BMMs. Neither isoform was expressed in unstimulated, Day 0 RAW macrophages or BMMs (Fig. 3B).

### 3.3 Characterization of knockdown cell lines

To determine the functional role of Ads during OCG, RAW macrophage clonal cell lines with reduced endogenous Ads expression were generated. To exclude the possibility of pleiotropic effects of shRNA expression or retroviral transduction, a control clonal cell line was also generated. In addition, WT macrophages were used as no infection controls.

**Figure 4:**
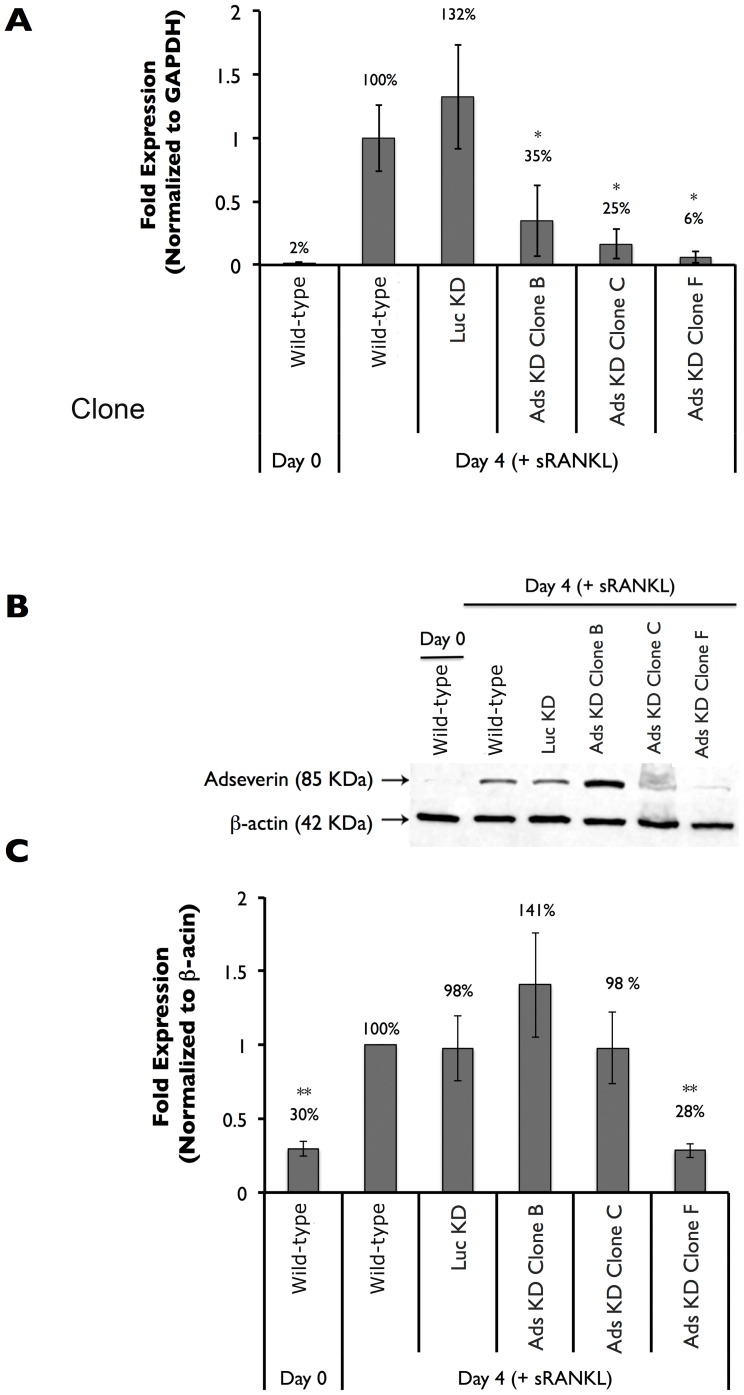
Adseverin knockdown clonal cell lines displayed varying degrees of Adseverin attenuation in response to sRANKL. **A**) Quantitative real-time PCR was used to quantify Ads gene expression in Day 0 and Day 4 osteoclast cultures. Results are expressed as fold expression versus WT Day 4 and are normalized against GAPDH ± SEM. Ads gene expression was not significantly different between Day 4 WT and Luc KD cultures. Ads transcript levels were significantly lower in all three Ads KD cell lines. Ads gene expression in Clones B and C were 35% and 25% that of Day 4 WT cultures, respectively. Ads KD Clone F cultures experienced the greatest reduction in Ads gene expression. Transcript levels in Day 4 Ads KD Clone F were similar to Day 0 WT cultures, which translated to 6% of Day 4 WT expression. **B**) Immunoblotting was used to quantify Ads protein expression in Day 0 and Day 4 osteoclast cultures. **C**) Results are quantified and expressed as fold expression versus WT Day 4 (±SD) and are normalized against β-actin. Ads protein expression was not significantly different between Day 4 WT, Luc KD, and Ads KD Clone B and C cultures. Ads KD Clone F cultures experienced the greatest reduction in Ads expression. Protein levels in Day 4 Ads KD Clone F were similar to Day 0 WT cultures, which translated to 28% of Day 4 WT expression (**p<0.01, n = 4).

Following expansion of the above mentioned clonal cell lines, quantitative real-time PCR was used to analyze the degree of Ads KD at the transcript level (Fig. 4A). Ads expression in WT Day 4 cultures was set at 100%. Ads gene expression in Day 0 WT cultures was 2% of Day 4 WT cultures. Ads gene expression was unchanged between Day 4 Luc KD and WT cultures. Day 4 osteoclasts derived from Ads KD Clone B, C and F cultures all experienced significant reductions in Ads expression. Ads transcript level in Day 4 cultures in Ads KD Clone B, C and F cells were 35%, 25% and 6% that of WT Day 4 cultures, respectively.

The degree of Ads attenuation was also determined at the protein level (Fig. 4B and 4C). Again, Ads expression in WT Day 4 cultures was set at 100%. Ads protein expression in Day 0, WT RAW macrophages was 30% of Day 4 WT cultures. Ads KD Clone B Day 4 cultures showed elevated (141%) albeit statistically non-significant increase in protein expression compared to Day 4 WT cultures. A slight, statistically non-significant, 2% reduction in protein expression was noted in Day 4 Ads KD Clone C and Luc KD cultures. Ads KD Clone F showed significant reductions in Ads protein levels after a 4-day stimulation with sRANKL. Ads protein expression in Day 4 Ads KD Clone F cultures was 28% that of the Day 4 WT cultures.

### 3.4 OCG is impaired in adseverin knockdown Clone F cells

**Figure 5:**
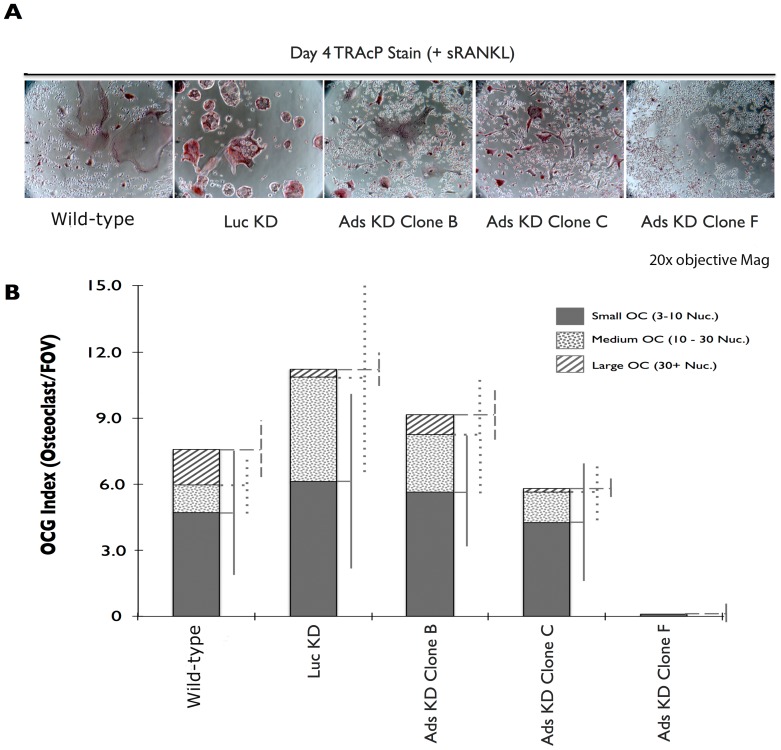
Adseverin knockdown Clone F displayed the greatest defect in osteoclast formation. **A**) 2.5×10^5^ RAW macrophages were seeded onto 6-well tissue culture plates and stimulated for 4 days with sRANKL. Representative photomicrographs of TRAcP stained osteoclasts were taken at 20× objective magnification. A profound lack of osteoclasts was observed in Ads KD Clone F. **B**) The number of osteoclasts per random field of view (OCG index) was quantified. The number of osteoclasts is represented as three distinct populations of small (3–10 nuclei/osteoclast, solid grey column), medium (5–7 nuclei/osteoclast, dotted column) and large (30+ nuclei/osteoclast, dashed column) osteoclasts (n≥10). Ads KD Clones B and C experienced marginal and statically non-significant reductions in the number of large osteoclasts when compared to WT cultures. No statistically significant difference in OCG was observed between the WT and Luc KD cultures. Ads KD Clone F macrophages failed for form multinucleated osteoclasts.

OCG was induced in WT, Luc KD, and Ads KD clonal cell lines via a four day stimulation with sRANKL. Cultures were routinely examined for DsRed signal using an inverted fluorescent microscope to ensure that presence of the shRNA cassette. As expected, only WT macrophages were DsRed negative (results not shown). There were no statistically significant differences between WT, Luc KD, Ads KD Clones B and C with respect to OCG index (Fig 5). All four populations had similar number of small, medium and large osteoclasts. Furthermore, the total number of osteoclasts in each genotype was not significantly different. Although not significant, a trend towards fewer large osteoclasts was noted in Ads KD Clones B & C (Fig. 5B). TRAcP stain intensity was reduced in Ads KD Clone F cultures in comparison to all other cell lines (Fig. 5A). The reduction in staining translated to virtually no multinucleated osteoclasts. Prolonging the culture by an additional two days did not increase the size or number of Ads KD Clone F osteoclasts (results not shown). Quantitative real-time PCR was used to ensure that Ads KD did not alter the expression of other vital genes during OCG. Gene expression of RANK, CD44, SIPRα and Gelsolin was unchanged between Ads KD Clone F and WT cells (Fig. S3).

### 3.5 Increased seeding density failed to rescue OCG defect in adseverin knockdown Clone F cells

**Figure 6:**
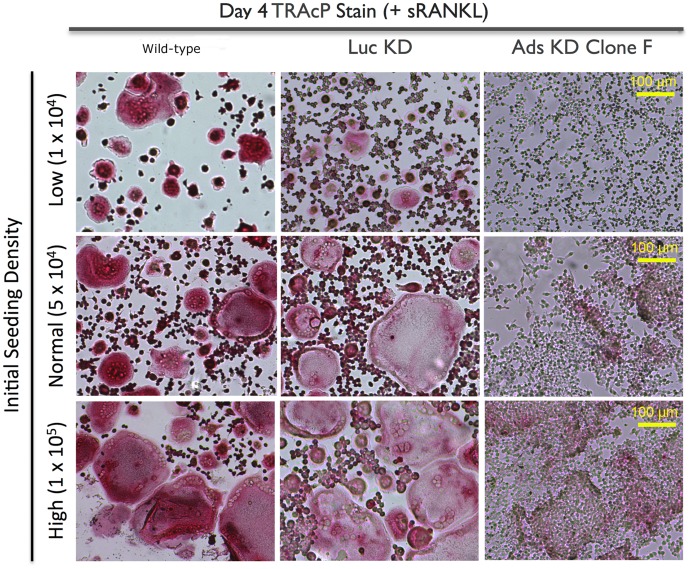
Increased seeding density failed to rescue OCG defect in Adseverin knockdown monocytes. WT, Luc KD and Ads KD (Clone F) monocytes were seeded at three initial plating densities in 12-well tissue culture plates, low (1×10^4^ cells/well), normal (5×10^4^ cells/well) and high (1×10^5^ cells/well). Cells were stimulated for 4 days with sRANKL to stimulate OCG. Representative TRAcP stained photomicrographs are shown. Even at the 10-fold-higher initial plating density, Ads-KD cells failed to form multinucleated osteoclasts, suggestive of a fusion defect.

OCG was induced and TRAcP positive osteoclasts were counted in Day 4 cultures (Fig. 6). OCG in WT and Luc KD macrophages was not affected by differential seeding density, as mature osteoclasts of all sizes were seen in Day 4 cultures. Increasing seeding density failed to fully rescue OCG in Clone F Ads KD progenitors, as Clone F cells failed to form TRAcP positive multinuclear osteoclasts regardless of initial seeding density. However, increased seeding density did seem to have a positive effect on TRAcP staining in the KD population. This observation is due to increased cell density and excessive non-specific staining of cellular debris as opposed to restoration of TRAcP expression.

### 3.6 Adseverin attenuation enhances migration in response to sRANKL

**Figure 7:**
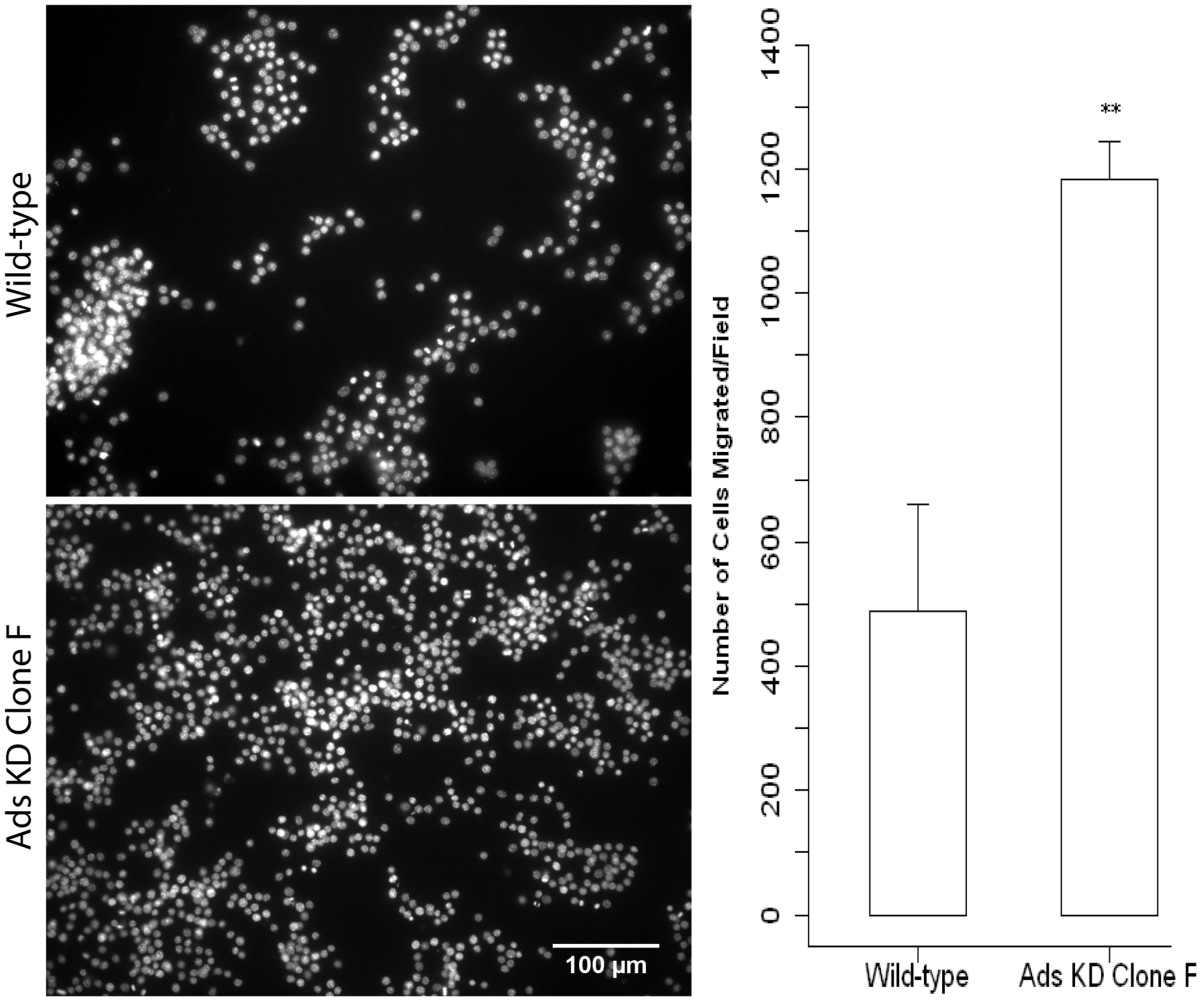
Adseverin knockdown osteoclast precursors display enhanced migration in response RANKL. 2×10^5^ Day 2 sRANKL starved WT and Ads KD (Clone F) cells were seeded in transwells (8 µm) pre-equilibrated in reduced DMEM containing 60 ng/mL sRANKL. The cells were starved for RANKL and then incubated for 20 hours to allow for migration (chemotaxis). After migration, the non-migratory cells on the top of the membrane were removed and migration assessed by enumerating the cells on bottom of the membrane (n = 3). Significantly more Ads KD Clone F cells migrated towards the chemokine sRANKL suggestive of enhanced migration ability in the absence of Ads.

Day 2 WT and Ads KD osteoclast cultures starved for sRANKL were incubated in transwell (8 µM) chambers conditioned with 60 ng/ml sRANKL, to analyze chemotaxis. Following the incubation period, Ads KD Clone F cells showed a statistically significant 2.4-fold increase in migratory cells when compared to WT cultures (Fig. 7).

### 3.7 Adseverin knockdown Clone F cells failed to resorb dentin discs substrates

**Figure 8:**
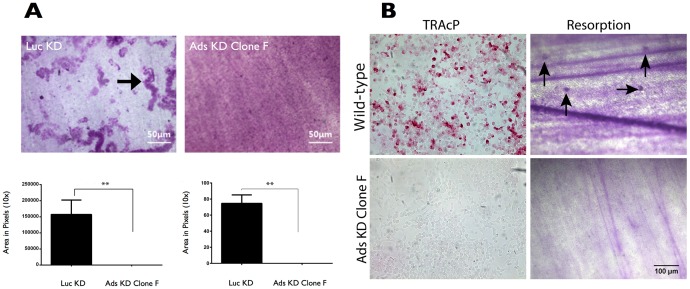
Adseverin knockdown osteoclasts fail to resorb mineralized matrix. **A**) Luc KD and Ads KD osteoclast precursors were cultured on dentin discs for 4 days. Mature Luc KD osteoclasts were capable of resorbing the dentin disc substrate (black arrow) significantly better than Ads KD cells, as indicated by the total resorbed area. Ads KD Clone F cultures had virtually no multinucleated osteoclasts or detectable resorption pits. **B**) WT and Ads KD (Clone F) cells were cultured for 2 days with sRANKL. More than 80% of WT cells were TRAcP. WT mononuclear TRAcP positive osteoclasts were capable of minimally resorbing the dentin slices as indicated by the small resorption pits (black arrows). Immature, Day 2 Ads KD cultures were TRAcP negative and displayed no resorptive ability.

The bone resorptive ability Day 4 Ads KD (Clone F) cultures were compared to Day 4 Luc KD cultures. Mature, Luc KD osteoclasts were capable of resorbing the dentin disc substrate at significantly higher rates than Ads KD cells (Fig. 8A, black arrow). Day 4 Ads KD Clone F osteoclast cultures had virtually no multinucleated osteoclasts, few mononuclear TRAcP positive osteoclasts and or detectable resorption pits (Fig. 8A). In order to assess the resorptive ability of premature, mononuclear osteoclasts, Day 4 Ads KD Clone F and Day 2 BMM-derived osteoclasts were also cultured on dentin discs. Nearly 80% of Day 2 WT mononuclear osteoclast precursors were TRAcP positive. In total 27±1.41 resorptive pits/12 mm^2^ disc were noted in Day 2 WT osteoclasts cultures, indicating that WT immature osteoclasts were capable of minimally resorbing dentin discs (Fig. 8B, black arrows). Conversely, no TRAcP staining or resorption pits were noted in Day 2 Ads KD Clone F cultures, indicating a lack of osteoclast differentiation and resorptive ability.

### 3.8 Adseverin knockdown disrupts F-actin architecture during OCG

**Figure 9:**
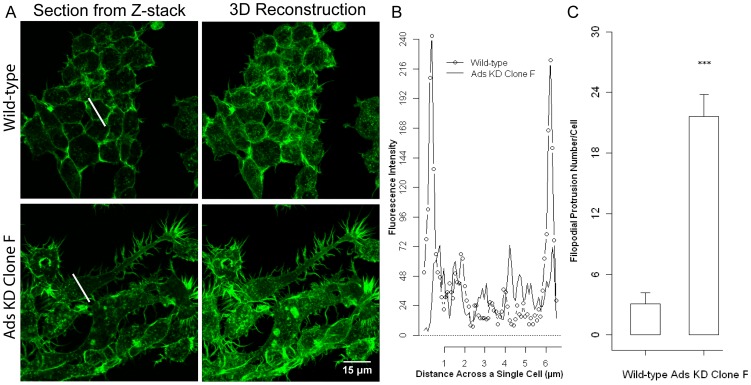
Adseverin knockdown alters F-actin architecture and cellular morphology in osteoclast precursors. **A**) WT RAW and Ads KD osteoclasts precursors were stimulated with sRANKL for two days, fixed and stained with green- fluorescent *phalloidin* to localize F-actin. **B**) F-actin intensity was plotted against the diameter of a cell (white line, Fig 9A). Well-defined cortical actin rings were noted in the periphery of WT cells as noted by high intensity fluorescence peaks at the cell boundaries, 0.5 µm and 6 µm. Fluoresce intensity across Ads KD cells was ill defined and lacked the characteristic peaks seen in the WT cells. Ads KD had a motile phenotype characterized by dense actin rich structure and significantly increased number of filopodial extensions at the cell periphery when compared to WT. **C**) The number of filopodial extensions of each cell type was determined. WT cells (leftmost column) had an average of 3 (+/−1) and Ads KD Clone F cells (rightmost column) had an average of 22 (+/−2) such membrane extensions.

F-actin, fluorescence intensity was plotted against the distance across a single cross section of a cell (Fig. 9A, white line). WT osteoclast precursors displayed well-defined cortical actin rings during the early phases of OCG as noted by high intensity fluorescence peaks at the cell boundaries, 0.5 µm and 6 µm (Fig. 9A & 9B). The KD of Ads protein expression resulted in significant morphological changes. The cortical actin rings commonly seen in the WT cells were lost in the Ads KD cells. The F-actin fluoresce intensity across Ads KD cells was ill defined and lacked the characteristic peaks seen in the WT cells (Fig. 9B). Dense F-actin islands and numerous actin projections resembling podosomes and filopodia characterized the F-actin architecture in the Ads KD cells. Although, fliopodial extensions were also noted in WT cells there was a significant 7-fold increase (Fig. 9C) in the Ads KD population giving the cells a more invasive and motile morphology (Fig. 9A).

### 3.9 Adseverin knockdown cell-rescue restores pre-osteoclast differentiation

**Figure 10:**
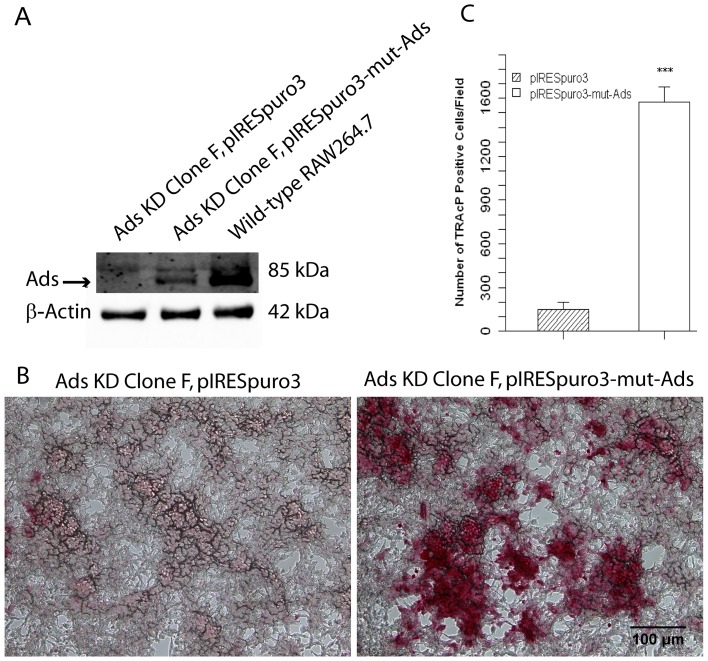
Adseverin knockdown cell-rescue restores pre-osteoclast differentiation. Two million Ads KD Clone F cells were transiently transfected with 2 µg of pIRESpuro3-mut-ads. The construct was constructed using site-directed mutagenesis such that the shRNA targeted sequence was mutated without changing the final protein product. A control transfection with an empty vector pIRES-puro3 was also carried out. **A**) Ads was not detected in the control transfection. Following transfection, Ads protein levels were increased to 22% of WT levels. **B & C**) The slight transient increase in Ads protein expression resulted in significantly more mononuclear, TRAcP positive ells. This phenotype was not found in the control transfection cultures.

A construct containing mutated Ads mRNA correlating to an Ads protein sequence identical to native Ads (pIRESpuro3-mut-Ads) was used to reintroduced Ads back into the Ads KD cells using transient transfections. An empty vector (pIRESpuro3-mut-Ads) was used as the control construct. Following transfections, the Ads protein expression level in cell lysates was measured after 4 days in culture (Fig. 10A). Ads was undetected in cell lysates from Ads KD Clone F transfected with the empty control vector. As expected these cells displayed a lack of OCG as noted by lack of TRAcP positive, multinucleated osteoclasts. Ads protein levels were slightly elevated (22% of WT) following transfection with the mutated Ads construct. The reintroduction of Ads was capable of restoring pre-osteoclast differentiation in the form of mononuclear TRAcP osteoclasts (Fig. 10B & C). It should be noted that no mature multinucleated osteoclasts were observed in culture following the transient reintroduction of Ads thereby eliminating the possibility of examining osteoclast function. The predominant reason for the lack of multinucleated osteoclasts is believed to be the transient nature of Ads rescue suggesting that Ads may be required for a prolonged period of time during OCG.

## Discussion

OCG consists of a series of coordinated cellular events including migration of fusion competent osteoclast precursors [43], cell-cell adhesion and membrane fusion[12]-[14]. Upon contact with the bone matrix, multinucleated osteoclasts become polarized, undergo distinctive cell spreading and allocate their nuclei to the anti-resorptive surface adhere to bone using actin rich structures and form a unique resorptive machinery [44], [45]. All of the events described above are heavily dependent on the dynamic regulation of the actin cytoskeleton. This clearly illustrates a potential functional role for the actin binding protein, Ads, in the regulation of osteoclast formation and function. Much of what is known about Ads was discovered through detailed examination of exocytosis in bovine chromaffin cells of the adrenal medulla. Immunocytochemistry analysis of chromaffin cells co-localize Ads with a cortical F-actin ring [24], [46], while numerous studies have demonstrated the role of Ads in depolymerizing the cortical F-actin ring and allowing for vesicular exocytosis [47]-[51]. To our knowledge, the microarray by Yang et al. [35] aimed at identifying genes differentially regulated by RANKL in mature osteoclasts was the first published report of Ads in the osteoclast biological system. Ads was up-regulated in mature osteoclasts and clustered with genes required for mature osteoclast attachment, movement and vesicular trafficking. This finding was validated in a microarray performed in our lab (Fig. 1), which identified Ads as a gene of interest during the early phases of OCG. Taken together, these reports prompted us to investigate the hypothesis that Ads is a RANKL induced regulator of OCG with a potential functional role both during the early phases of OCG and in mature osteoclasts.

The temporal expression pattern of Ads was suggestive of a potential role during the earlier phases of OCG with a potential role in mature osteoclasts (Fig. 2). Further, it appears that any role for Ads D5 during OCG would be very limited, as only the full-length isoform of Ads was up-regulated during OCG in response to sRANKL (Fig. 3). To address the functional role of Ads during OCG, RNAi gene silencing was used to generate clonal Ads KD cell lines. The viral transduction and shRNA expression did not have any non-specific effects on OCG as indicated by the similarities between the non-infected WT and control, Luc KD cultures (Figs 4-6, 8, S3). Ads KD Clone F experienced the greatest attenuation of Ads expression and the most distinct phonotypical variation from WT cells (Fig. 5) and was chosen as the experimental cell line for further experiments.

Considering the structural and functional similarities between Ads and Gelsolin, it is plausible to assume that these two proteins play similar or even redundant roles during OCG. Gelsolin, which has been implicated in osteoclast matrix adhesion, actin remodeling, motility and bone resorption [52] showed no changes in expression during OCG in response to RANKL. Ads on the other hand was significantly up-regulated in the early phases of OCG. Based on the differences in expression patterns, it is more likely that Ads and Gelsolin have divergent roles in the regulation of osteoclast formation and function. Strong evidence towards the divergent roles of Ads and Gelsolin during OCG comes from findings of Cellaiah et. al [52], where it was demonstrated that Gelsolin was not required for osteoclast precursor fusion or osteoclast formation. Instead, Gelsolin null mice experienced abnormal actin cytoskeleton characterized by a loss of podosome assembly, reduced osteoclast motility caused by decreased podosome turnover, which contributed to decreased *in vivo* bone resorption.

The depletion of endogenous Ads (Clone F) resulted in a lack of multinucleated, TRAcP positive osteoclasts (Fig. 5), indicating a potential defect in early phases of OCG. The early phases of OCG can be divided into induction of fusion competency [9], migration [10]-[12] and membrane fusion [14]. A differential seeding density experiment was used as an indirect assay to tease out migration and/or fusion deficiencies in Ads KD osteoclast precursors. The rationale behind the differential seeding density experiments was as follows: 1) If fusion was compromised while migration was unaffected in Ads KD cell line, then OCG would be defective regardless of cell proximity; 2) If migration was compromised while fusion was unaffected in the Ads KD cell line, then the migration defect would be simply overcome by the increased initial proximity of precursors, resulting in large multinucleated osteoclasts at high densities; and 3) In the case of a concurrent defect in migration and fusion, this assay is incapable of determining the extent of the migration defect, but will still result in a lack of multinucleated osteoclasts due to a fusion defect. Large, TRAcP positive, multinucleated osteoclasts were observed regardless of seeding density in WT and Luc KD cell lines, while the phenotype of multinucleated osteoclasts was not rescued by increasing seeding density in the Ads KD (Clone F) cells (Fig. 6). Therefore, our findings favor the first or third possibilities, indicating a potential fusion defect in the Ads KD (Clone F) osteoclast precursors. As mentioned before, the seeding density assay was unable to determine the extent of migration ability of the KD cells. However, pronounced cellular clumps observed within two days of differentiation at low and normal densities (results not shown) and transwell migration assays (Fig. 7) suggest enhanced migratory ability in KD cell lines. Further evidence supporting enhanced migratory of Ads KD cells ability comes from experiments that demonstrated the effect of Ads attenuation on morphological changes in cell shape and sub-cellular localization of F-actin (Fig. 9). Decreased Ads expression equated to the loss of cortical actin rings found in WT cultures. This was a surprising finding, as in most other cell systems, Ads induction results in the depolymerization of the cortical actin ring immediately prior to exocytosis. Therefore, a logical expectation would be to find dense, well-defined cortical actin rings in the absence of Ads. However, in the absence of Ads, the F-actin cytoskeleton is reorganized into dynamic F-actin rich structures resembling filopodia, and podosome cores. This finding corroborates with the finding of enhanced mobility and migration of Ads KD cells. Osteoclast migration is mediated through podosomes [53], while osteoclasts gain the ability to transmigrate through cell layers by arranging their cytoskeleton into long protrusive projections reminiscent of invadopodia [54]. The increased filopodia formation in the absence of Ads, is suggestive of a regulatory change in actin reorganization from fusion to migration.

As previously stated, the lack of multinucleated osteoclasts in the KD cell line implicates a fusion defect. That being said, a fusion defect does not equate to a defect in OCG [55], [56]. Mononuclear, TRAcP positive osteoclasts are still capable of functioning and resorbing bone-like substrates, albeit less efficiently than multinucleated osteoclasts. This was clearly demonstrated by small resorption pits in dentin slices created by mononuclear TRAcP positive osteoclasts seen in two-day WT cultures (Fig. 8B). However, in the absence of Ads, there is also a marked defect in OCG, as noted by the lack TRAcP cells even after a four-day stimulation. As expected, the few TRAcP positive mononuclear osteoclasts noted in Ads KD (Clone F) cultures failed to resorb bone to any significant degree (Fig. 8). Therefore, in addition to a potential role in regulating fusion, Ads may also play a regulatory role during the mononuclear stages of OCG prior to pre-osteoclast fusion. It is unclear if the lack of fusion prevents differentiation or if altered differentiation program prevents fusion events. In order to illustrate the specific effects of targeted Ads attenuation, a mutated, shRNA insensitive Ads construct with altered message yet identical protein sequence was generated and used to transiently rescue the Ads defect in the KD cells (Fig. 10). Following the transient reintroduction of Ads, we were capable of rescuing TRAcP expression but not the phenotype of multinucleated osteoclasts. This observation can partly be explained by the transient nature of the rescue experiments, which only accommodate a 48-hour peak in Ads protein expression 24 to 48 hours post transfection. Earlier experiments have shown that although Ads expression peaks after two days of sRANKL stimulation, Ads levels remained elevated during OCG and in mature osteoclasts. Due to technical difficulties of stable transfection, we could not stably re-introduce Ads into the KD system. However, the transient rescue experiments are promising evidence depicting the importance of Ads for osteoclast differentiation. Presumably, mature, multinucleated osteoclast phenotype could be rescued if higher levels of Ads could be reintroduced into the system for a longer period of time. Taken together, we can conclude that Ads is critical in the regulation of *in vitro* OCG both in terms of differentiation and pre-osteoclast fusion. One possible mechanistic explanation is that in the absence of Ads, osteoclast precursors shift actin cytoskeletal organization to favor migration and inhibit fusion, resulting in a marked decrease in the number of multinucleated, TRAcP functioning osteoclasts. Further, the lack of Ads may have wider reaching implications downstream of the RANKL-RANK signal cascade resulting in altered osteoclast differentiation. The preliminary nature of this study has not focused on the intricate signaling cascade downstream of the RANKL-RANK in the Ads KD system. That being said, this proposed role of Ads in the regulation of osteoclast differentiation is consistent with the previously described cellular differentiation of role of Ads in platelet maturation and chondrocyte differentiation [29], [30]. As such, we can conclude that a threshold, basal level of Ads may be required during the early mononuclear stages of OCG to promote TRAcP expression and osteoclast differentiation. Further, it is plausible that during the later stages of OCG, when cell-cell fusion is necessary to allow for the formation of large multinucleated osteoclasts, prolonged elevated levels of Ads expression may be required for the intricate regulation of pre-osteoclast fusion.

This project has already taken major strides towards determining the role of Ads in the osteoclast biological system. We have described the temporal expression pattern of Ads during OCG in response to RANKL and we have shown how attenuated Ads expression has deleterious impacts on the pre-osteoclast differentiation and the fusogenic ability of pre-osteoclasts. However, there are a number of important questions that still remain unanswered. First and foremost, we have yet to decipher the mechanism of Ads mediated osteoclast progenitor fusion. Alternatively, we plan to characterize the *in vivo* skeletal effect of Ads using newly developed transgenic mice containing a floxed Ads allele crossed this with LysM Cre transgenic mice to create viable conditional knockouts, in which Ads is deleted in osteoclast precursors and osteoclasts. Lastly, we plan on generating several Ads constructs with deletions of domains for either actin severing, actin nucleation or actin capping, to determine the mechanisms through which domain(s) regulates actin mediated pre-osteoclast fusion. All in all, the evidence presented in this paper represents an advancement in osteoclast biology. For the first time the role of Ads in osteoclast differentiation has been examined. Furthermore, although not unique to osteoclasts, Ads may also serve as a new marker for osteoclast differentiation.

## Supporting Information

Figure S1 **Detection of Adseverin D5 using primers flanking the 5^th^ Domain.** A schematic representation of the pSE380 bacterial expression vector with the full-length Ads (pSE380-A-FL) and Ads D5 (pSE380-A-D5) isoforms within its Nco I and Pst I sites. Primers flanking the 5^th^ domain of Ads (esD5 forward and esD5 reverse) allow for the amplification of a 650 bp amplicon from Ads full-length plasmid and a 350 bp amplicon of the Ads D5 plasmid.
(TIFF)

Figure S2 **Schematic of knockdown clonal cell lines.** Viral supernatant with the RNAi-Ready pSIREN-RetroQ-DsRed Express vector containing the Ads or Luc KD shRNA sequence was harvested and used to infect RAW macrophages. Infected cells were sorted based on the DsRed signal by fluorescence-activated cell sorting and subjected to a limiting dilution to obtain clonal cell lines. Colonies were examined under an inverted fluorescent microscope and DsRed positive colonies were identified and expanded. A single Luc KD clonal cell line (Luc KD) and three Ads KD clonal cell lines (Clones B, C, & F) were generated. WT RAW macrophages were used as non-infection controls. The schematic above shows that only cells stably infected cells were DsRed positive, while the WT cells showed no DsRed signal.
(TIFF)

Figure S3 **Adseverin knockdown does not alter the expression of multiple genes important in osteoclastogenesis.** Quantitative real-time PCR was used to quantify gene expression on Days 0 and 4 of osteoclast cultures. Results are expressed as fold expression versus GAPDH used as internal control. There were no statistically significant differences in transcript levels of RANK, SIRPα, CD44 and Gelsolin between Ads KD and WT Day 0 and Day 4 osteoclast cultures (n = 3).
(TIFF)
