# Supplementary figures and images for: The Actin Binding Protein Adseverin Regulates Osteoclastogenesis
Source: PLoS One. 2014 Oct 2;9(10):e109078. doi: 10.1371/journal.pone.0109078 (PMC4183545; doi:10.1371/journal.pone.0109078)

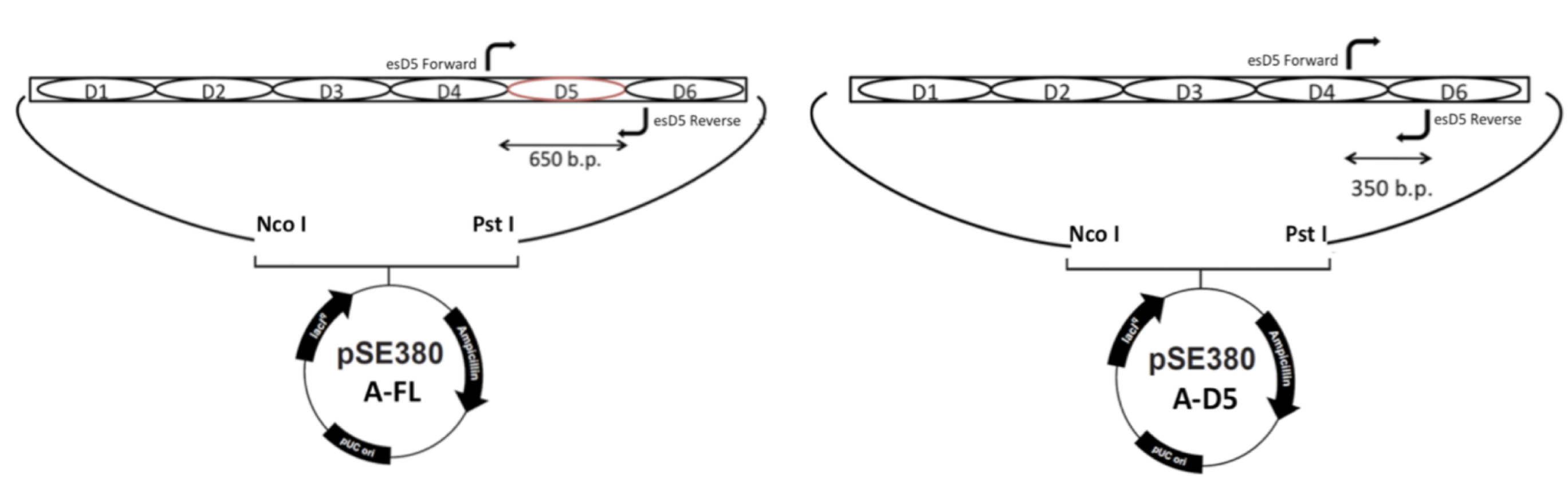

Supplement: Figure S1 — Detection of Adseverin D5 using primers flanking the 5th Domain. A schematic representation of the pSE380 bacterial expression vector with the full-length Ads (pSE380-A-FL) and Ads D5 (pSE380-A-D5) isoforms within its Nco I and Pst I sites. Primers flanking the 5th domain of Ads (esD5 forward and esD5 reverse) allow for the amplification of a 650 bp amplicon from Ads full-length plasmid and a 350 bp amplicon of the Ads D5 plasmid. (TIFF) [file pone.0109078.s001.tiff]

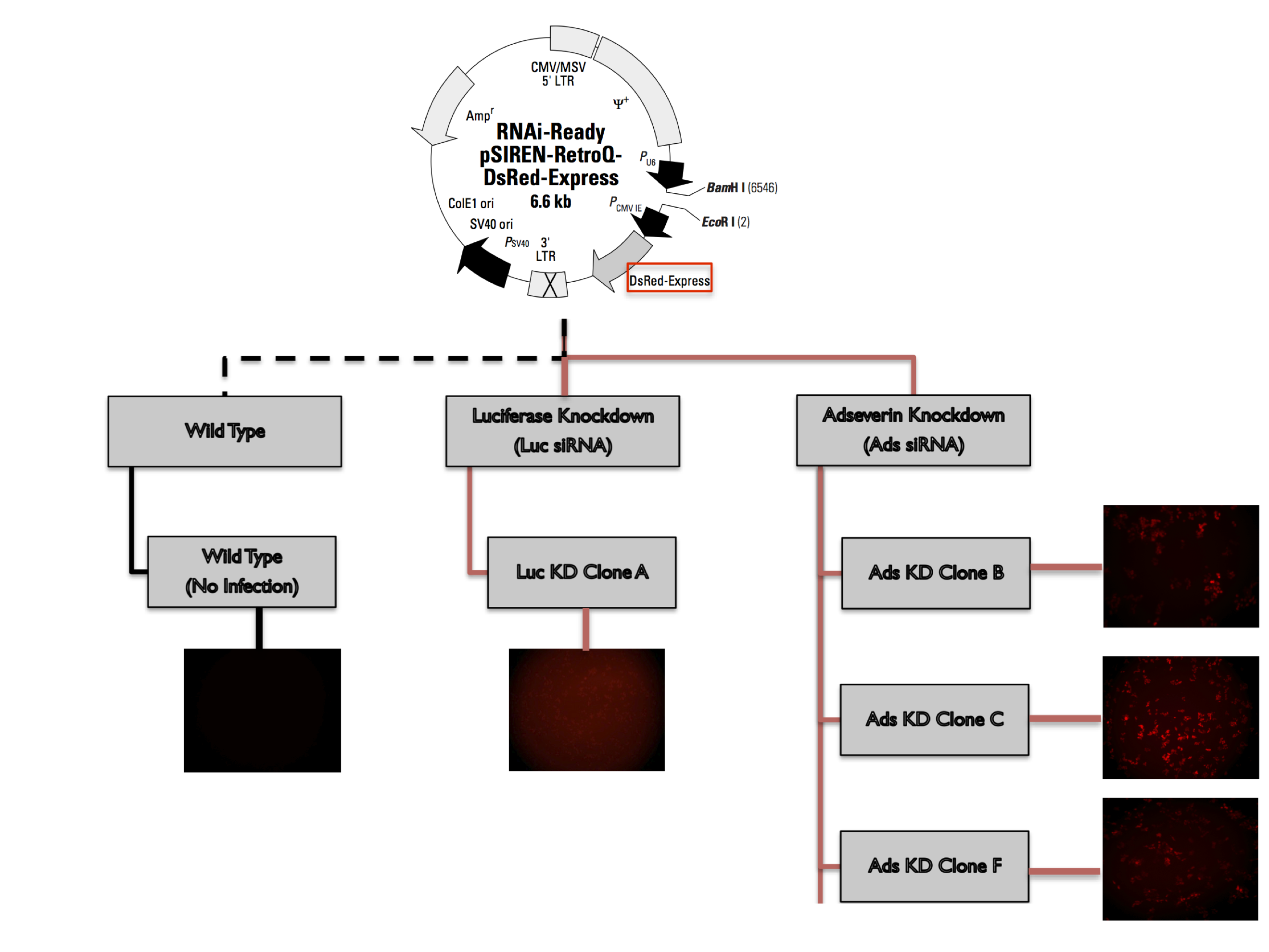

Supplement: Figure S2 — Schematic of knockdown clonal cell lines. Viral supernatant with the RNAi-Ready pSIREN-RetroQ-DsRed Express vector containing the Ads or Luc KD shRNA sequence was harvested and used to infect RAW macrophages. Infected cells were sorted based on the DsRed signal by fluorescence-activated cell sorting and subjected to a limiting dilution to obtain clonal cell lines. Colonies were examined under an inverted fluorescent microscope and DsRed positive colonies were identified and expanded. A single Luc KD clonal cell line (Luc KD) and three Ads KD clonal cell lines (Clones B, C, & F) were generated. WT RAW macrophages were used as non-infection controls. The schematic above shows that only cells stably infected cells were DsRed positive, while the WT cells showed no DsRed signal. (TIFF) [file pone.0109078.s002.tiff]

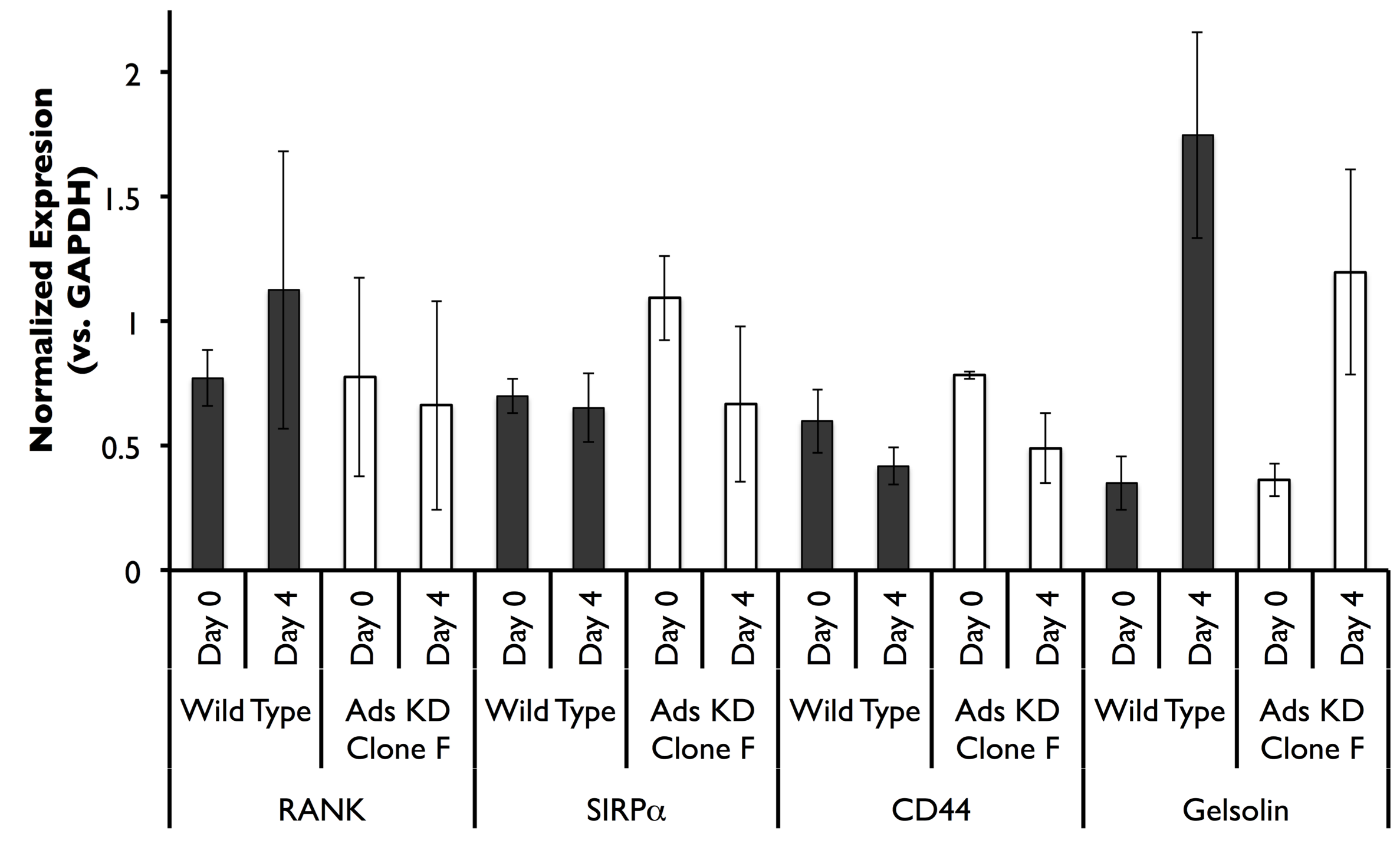

Supplement: Figure S3 — Adseverin knockdown does not alter the expression of multiple genes important in osteoclastogenesis. Quantitative real-time PCR was used to quantify gene expression on Days 0 and 4 of osteoclast cultures. Results are expressed as fold expression versus GAPDH used as internal control. There were no statistically significant differences in transcript levels of RANK, SIRPα, CD44 and Gelsolin between Ads KD and WT Day 0 and Day 4 osteoclast cultures (n = 3). (TIFF) [file pone.0109078.s003.tiff]
